# Observation of SAM-VI Riboswitch Dynamics Using Single-Molecule FRET

**DOI:** 10.3390/biom15060841

**Published:** 2025-06-09

**Authors:** Yanyan Xue, Yi Sun, Yichun Xia, Xiuming Liu, Hua Dai

**Affiliations:** 1 Institute of Translational Medicine, School of Medicine, Yangzhou University, Yangzhou 225001, China; 232210231@stu.yzu.edu.cn (Y.S.); 232210241@stu.yzu.edu.cn (Y.X.); 232210213@stu.yzu.edu.cn (X.L.); 2 The Key Laboratory of the Jiangsu Higher Education Institutions for Nucleic Acid & Cell Fate Regulation (Yangzhou University), Yangzhou 225001, China; 3 State Key Laboratory of Microbial Metabolism, School of Life Sciences and Biotechnology, Shanghai Jiao Tong University, Shanghai 200240, China

**Keywords:** SAM-VI riboswitch, dynamic structure, PLOR, fluorescent labeling, smFRET

## Abstract

Riboswitches regulate gene expression through intricate dynamic conformational transitions, with divalent cation Mg^2+^ and their ligands playing pivotal roles in this process. The dynamic structural mechanism by which the S-adenosyl-L-methionine (SAM) responsive SAM-VI riboswitch (riboSAM) regulates the downstream SAM synthase gene translation remains unclear. In this study, we employed position-selective labeling of RNA (PLOR) to incorporate Cy3-Cy5 into designated positions of riboSAM, applying single-molecule Förster resonance energy transfer (smFRET) method to track its conformational switches in response to Mg^2+^ and SAM. smFRET analysis revealed that in the absence of Mg^2+^ and ligand, riboSAM predominantly adopted a translation-activating *apo* conformation. Physiological concentrations of Mg^2+^ induced riboSAM to fold into dynamic *transit-p* and *holo-p* states, creating a transient and structurally pliable binding pocket for ligand binding. SAM binding locks the dynamic *transit-p* and *holo-p* states into their final stable *transit* and *holo* conformations through conformational selection, turning off downstream cis-gene expression and completing feedback regulation of cellular SAM concentration. The observed synergistic regulatory effect of Mg^2+^ ions and ligand on riboSAM’s conformational dynamics at single-molecule resolution provides new mechanistic insights into gene regulation by diverse riboswitch classes.

## 1. Introduction

Riboswitches are widespread RNA cis-regulatory elements located within the 5′-untranslated regions (UTRs) of bacterial mRNAs, playing key roles in the regulation of gene expression [1,2,3]. A typical riboswitch consists of a highly structured aptamer domain and a downstream adjoining expression platform. The aptamer domain functions as a sensor, binding its cognate ligand with high selectivity and specificity. This binding induces a conformational switch in the expression platform, mediating downstream gene expression at the level of transcription, translation, or RNA processing [4,5]. The proteins encoded by riboswitch-regulated genes are typically involved in the production or transport of the small metabolite that binds to the riboswitch. Consequently, the recognition and binding of a riboswitch to its own metabolite acts as a feedback mechanism to regulate gene expression. So far, more than 40 classes of riboswitches have been identified in nature, sensing numerous metabolites including enzyme cofactors, nucleotide derivatives, amino acids, metal ions, etc. [6,7,8,9,10,11]. Although the expression platforms vary greatly, the conserved features of the aptamer domain and the cognate ligand identity have been used to differentiate various riboswitch classes [6,7].

**Figure 1:**
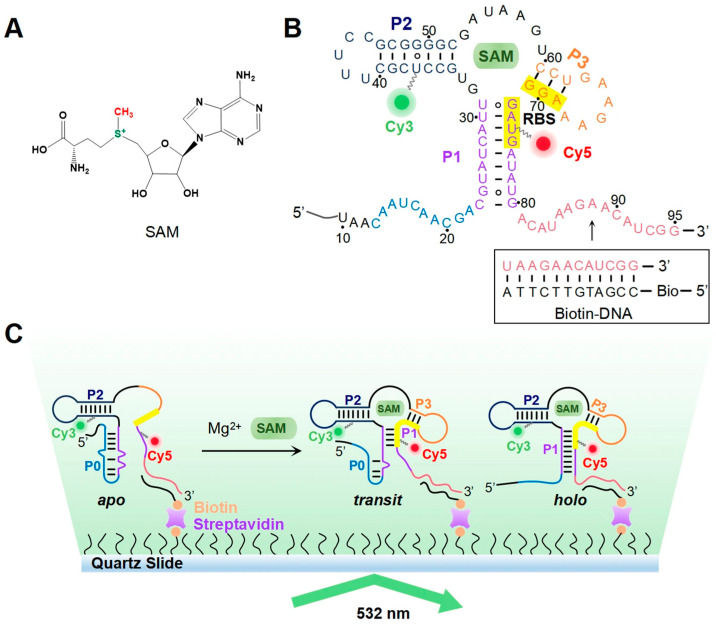
A schematic illustration of the smFRET study of riboSAM. (**A**) The chemical structure of SAM. (**B**) The secondary structure of the *holo* state of 37Cy3-74Cy5-riboSAM. The positions of Cy3 and Cy5 are shown as green and red balls, respectively. The sequences of the P0 and P1 helices  are shown in blue and purple, respectively. The nucleotides in the RBS are highlighted in yellow. The last 12 nucleotides (nt) at the 3′ end of 37Cy3-74Cy5-riboSAM (shown in red) are used to hybridize with a biotinylated DNA oligo, enabling immobilization of the RNA molecule onto a smFRET slide. (**C**) Experimental smFRET setup.

SAM, a central metabolite that acts as a co-factor of various enzymatic reactions in all living organisms [12,13,14], is enzymatically produced by SAM synthetase using ATP and methionine. The chemical structure of SAM contains a positively charged sulfonium center substituted by a methyl, an aminocarboxypropyl and a 5′-adenosyl group (Figure 1A). The importance of SAM lies in its use as a methyl group donor for methyltransferases, which transfer its methyl group to diverse acceptor substrates, including nucleic acids, proteins, biologic amines, phospholipids, and various small molecules [13]. Given the importance of methylation in myriad cellular processes, the intracellular concentration of SAM is strictly regulated. In bacteria, SAM synthesis is tightly controlled by SAM riboswitches, which represent the most abundant class of riboswitches in bacteria [15,16,17]. SAM-VI is a new subclass of SAM riboswitches that are predominantly found in the 5′-UTR of the mRNA encoding SAM synthetase in *Bifidobacterium* [18,19,20,21]. On recognition of excessive levels of cellular SAM, they turn off the translation of the downstream gene encoding SAM synthetase, thereby maintaining SAM homeostasis. Crystallographic data show that the *Bifidobacterium angulatum* SAM-VI riboswitch (lacking the P0 sequence) adopts a three-helix structure comprising P1, P2, and P3 upon ligand binding, with a ligand-binding pocket formed at the junction of these helices [19]. Nuclear magnetic resonance (NMR) and co-transcriptional dynamic structural studies have shown that the full-length *Bifidobacterium breve* SAM-VI riboswitch (riboSAM) (including the P0 sequence) can form five distinct conformations under different SAM conditions: *apo*, *transit-p*, *transit* (*transit-p* binds with SAM), *holo-p* and *holo* (*holo-p* binds with SAM) (Figure 1B,C and Figure S1) [20]. The competitive formation of the P0 and P1 helices, driven by their shared sequence segment, signifies the emergence of distinct conformations for each. Specifically, the formation of the P0 helix stabilizes the *apo* state, exposing the ribosome binding site (RBS) and turning translation of the downstream cis-gene to ‘ON’. In contrast, the formation of the P1 helix results in the *holo*/*holo-p* states, which prevents ribosome binding to the RBS and inhibits translation. The intermediate conformations between *apo* and *holo*/*holo-p*, namely *transit*/*transit-p*, form a unique bifurcated helix characterized by the partial unzipping of the P0 helix and the partial formation of the P1 helix (Figure 1C and Figure S1). *Transit* state also inhibits gene translation, enabling riboSAM to efficiently respond to changes in intracellular SAM concentration with less energy consumption than *holo* conformation [20].

Divalent cations play important roles in the folding and stabilization of complex RNA structures [22,23]. An abundance of negatively charged phosphate groups along the RNA phosphodiester backbone creates electrostatic repulsion, which can hinder the formation of compact three-dimensional (3D) structures of RNA. This anisotropic electrostatic interaction is particularly unfavorable for RNA folding. Consequently, divalent metal ions, especially Mg^2+^, with an optimal charge-to-size ratio [24,25], play a critical role in RNA folding. Mg^2+^ can stabilize the final RNA structure through directly coordinating with negatively charged groups of RNA or in water-mediated interactions with hydrated Mg^2+^ ions (Mg(H_2_O)_6_^2+^). Mg^2+^ ions also affect the folding rate, stabilize folded intermediates, or destabilize misfolded conformations of RNA [24,26]. Riboswitches undergo intricate folding processes to adopt the essential 3D conformations essential for the recognition and binding of specific ligands, wherein divalent cations play a pivotal role [27,28]. X-ray crystallographic studies have identified Mg^2+^ ions binding sites in numerous riboswitches, including the TPP riboswitch, adenine riboswitch, and SAM-VI riboswitch [19,29,30], etc. In previous studies examining riboSAM’s co-transcriptional folding dynamics, we employed a physiologically relevant Mg^2+^ concentration (2 mM) matching the intracellular range observed in bacteria (0.5–4 mM) [20,22,23,27]. However, there is a paucity of structural information for Mg^2+^-free riboSAM conformations, and a dearth of structural detail on Mg^2+^-induced folding of riboSAM. To elucidate the role of Mg^2+^ in riboSAM folding and its conformational dynamics during ligand recognition, we used the PLOR method [31,32] to incorporate the fluorophores Cy3 and Cy5 into strategic positions within riboSAM (Cy3-Cy5-riboSAM). Subsequently, we employed smFRET technology to monitor the structural transitions of Cy3-Cy5-riboSAM under varying Mg^2+^ concentrations and ligand conditions (Figure 1C). Our results show that, in the absence of both Mg^2+^ and ligand, riboSAM primarily adopts a translation-activating *apo* conformation. Physiological concentrations of Mg^2+^ induce riboSAM to fold into dynamic *transit-p* and *holo-p* conformations, while the ligand SAM locks these dynamic conformations into their final stable *transit* and *holo* states. These conformational changes sequester the RBS, thereby inhibiting translation of the downstream cis-gene and completing feedback regulation of SAM level.

## 2. Materials and Methods

### 2.1. Preparation of DNA Templates for PLOR Reaction

The DNA and RNA sequences used for riboSAM synthesis are listed in Table S1. The biotin–DNA templates used in PLOR were produced by PCR with biotin and 2′-O-methylguanosine (mG) groups at the 5′-ends of the coding and template strand, respectively. The biotin enables immobilization of DNA templates onto streptavidin-coated agarose beads (Smart-Life sciences, Changzhou, China), facilitating convenient reagents and buffers exchange through filtration during PLOR. The mG modification was used to minimize non-template transcripts. The DNA templates were purified by 10% urea-polyacrylamide gel electrophoresis (PAGE) as previously described [31,32] and then incubated with streptavidin-coated agarose beads at 4 °C overnight to immobilize the DNAs to the beads, generating solid-phase beads-DNA for use as the DNA template in PLOR reaction.

### 2.2. Preparation of Unlabeled riboSAM

Unlabeled riboSAM was synthesized via in vitro transcription using PCR-generated DNAs as the template. The transcription reaction was performed at 37 °C for 3–6 h in a buffer containing 40 mM HEPES (pH 8.0), 10 mM DTT, and 28 mM MgCl_2_, with a 0.02–0.1 µM DNA template, 0.01–0.05 µM T7 RNA polymerase, and 5 mM NTPs. The RNA products were purified by 12% urea-PAGE, then exchanged into the desired buffer and stored at −80 °C prior to use.

### 2.3. Preparation of 37Cy3-74Cy5-riboSAM

PLOR was used to synthesis 37Cy3-74Cy5-riboSAM in a 12-step reaction. A detailed description is listed in Table S2. In the first step, a mixture containing 1 mL of 10 µM beads-DNA, equimolar T7 RNA polymerase, 1.12 mM ATP, 0.96 mM GTP, and 32 µM UTP in the initiation buffer (40 mM Tris-HCl, pH 8.0, 100 mM K_2_SO_4_, 6 mM MgSO_4_, 10 mM DTT) was incubated at 37 °C for 15 min, yielding the 12-nt riboSAM fragment. Filtration and bead-rinsing were then performed at least three times using a washing buffer (40 mM Tris-HCl, pH 8.0, 6 mM MgSO_4_) to remove the residual NTPs. Unless otherwise specified, the filtration and bead washing were conducted between each step. For step 2, the transcription was performed in the elongation buffer (40 mM Tris-HCl, 6 mM MgSO_4_, 10 mM DTT, pH 8.0) containing 40 µM ATP, 30 µM CTP, and 10 µM UTP at 25 °C for 10 min. Subsequent steps followed the same protocol but with different NTP mixtures (Table S2). Steps 6 and 11 were conducted at 30 °C to enhance Cy3/Cy5-UTP (Figure S2) (APExBIO Technology, Houston, TX, USA) incorporation efficiency. The final RNA products at step 12 were purified sequentially by 12% urea-PAGE and reverse-phase high-performance liquid chromatography (RP-HPLC) using a C8 column (Phenomenex Luna, Torrance, CA, USA).

The purified 37Cy3-74Cy5-riboSAM was detected by RP-HPLC with the following procedure: the initial 2 min was with 10% buffer B (75% acetonitrile, 100 mM TEAA) and 90% buffer A (DEPC-H_2_O with 100 mM TEAA), followed by a 50 min linear gradient from 10% to 70% buffer B at a flow rate of 0.3 mL/min. Detection was performed under both UV (260 nm) and fluorescence (550 or 650 nm) irradiation.

### 2.4. smFRET Detections

A total of 0.01 mg/mL streptavidin (Invitrogen^TM^, Carlsbad, CA, USA) in T50 buffer (10 mM Tris-HCl, 50 mM NaCl, pH 8.0) was injected into the flow chambers on the biotin-polyethylene glycol functionalized glass slides, incubated for 5 min, and subsequently washed with T50 buffer. The last 12 nucleotides at the 3′ end of 37Cy3-74Cy5-riboSAM are complementary to a biotinylated DNA oligo, enabling immobilization of the RNA molecules onto the slides for the smFRET study. Between 50 and 100 pM 37Cy3-74Cy5-riboSAM samples were immobilized in the flow chambers. After 5 min of immobilization, the unbound molecules were washed away from the chambers by imaging buffer (10 mM Tris-HCl, 50 mM NaCl, 0–10 mM MgCl_2_, 0–0.5 mM SAM, pH 8.0). smFRET data were collected in the imaging buffer containing an oxygen scavenger system: 5 mM protocatechuic acid (Aladdin Bio-Chem Technology Co., Shanghai, China), 100 nM protocatechuate dioxygenase, and 3 mM Trolox (MCE, Monmouth Junction, NJ, USA) to alleviate photobleaching and blinking of Cy3 and Cy5.

All smFRET measurements were performed using an objective-type total internal reflection fluorescence (TRIF) microscopy based on a Nikon Eclipse Ti inverted microscope (Nikon, Tokyo, Japan) at 25 °C. The movies were acquired over 100 s using an EMCCD camera (Andor iXon Ultra 897, Andor Technology, Belfast, UK) with a temporal resolution of 100 ms per frame. The imaging system employed two solid-state lasers (OBIS Smart Lasers, Coherent Inc., Santa Clara, CA, USA) at 532 nm and 640 nm for excitation, with laser modulation synchronized to the camera via digital triggering signals. Cy3 in 37Cy3-74Cy5-riboSAM were continuously excited using the 532 nm laser line. Total internal reflection fluorescence (TIRF) microscopy was performed using a high numerical aperture oil immersion objective (100×, NA 1.49, Apo TIRF, Nikon, Tokyo, Japan) to generate an evanescent wave for surface-selective illumination. Fluorescence trajectories of individual molecules were recorded at a frame rate of 10 Hz.

### 2.5. Quantification and Statistical Analysis of smFRET Data

Locations of molecules and fluorescence intensity traces for each molecule were extracted from raw movie files using the open-source iSMS software (Version 2.01) [33]. Fluorescent spots were identified by fitting two-dimensional Gaussian functions on the EMCCD after subtracting the background signals. Single-molecule trajectories and photobleaching events were automatically identified using deepFRET software (Version 3) [34], with manual screening sometimes required to enhance analysis accuracy. For subsequent statistical analysis, we selected trajectories exhibiting characteristic anticorrelated donor-acceptor intensity changes prior to photobleaching. The FRET efficiency (*E_FRET_*) was calculated using the standard equation:

*E_FRET_* = *I_A_*/(*I_A_* + *I_D_*)

where *I_A_* and *I_D_* represent acceptor (Cy5) and donor (Cy3) intensities, respectively. An approximate distance between Cy3 and Cy5 was calculated by employing the equation *E_FRET_* = 1/(1 + (*R*/*R*_0_)) [35,36], utilizing a Förster radius (R_0_) value of 55 Å. An idealized representation of the FRET efficiency was obtained by employing the hidden Markov modeling (HMM) package vbFRET, which utilized an empirical Bayesian approach to estimate the FRET states and the time points of transitions [37]. The transition events were counted by referencing the state sequence assigned by HMM, and these events were subsequently visualized through transition occupancy density plot (TODP) using the Python module matplotlib (Version 3.5.2). The lifetimes were plotted in histogram and fitted with a simple exponential decay function by Origin 8.5 software to obtain the rate constants.

## 3. Results

### 3.1. Synthesis and Characterization of Site-Specific Cy3-Cy5-Labeled riboSAM

The full-length SAM-VI riboswitch used in this work, riboSAM, was derived from *Bifidobacterium breve*, which contains both the P0 and P1 helix sequences (Figure 1B,C). Previous studies of the folding of riboSAM into its 3D conformation have shown that the spatial distance between the U37 site in the P2 helix and the U74 site in the P1 helix is approximately 26 Å [20]. Therefore, by specifically labeling these two sites with Cy3 and Cy5, smFRET can be used to differentiate the *apo*, *transit*/*transit-p*, and *holo*/*holo-p* conformations, which display distinct FRET efficiencies of ~0.2, 0.6, and 0.8, respectively [20]. In this study, we also labeled the U37 and U74 sites of riboSAM with Cy3 and Cy5 to generate 37Cy3-74Cy5-riboSAM (Figure S3), and used smFRET to investigate its structural dynamics in response to Mg^2+^ ions and the ligand SAM. The 37Cy3-74Cy5-riboSAM molecules were synthesized using a 12-step PLOR reaction (Figure S3), purified by RP-HPLC equipped with a C8 column. In the RP-HPLC, 37Cy3-74Cy5-riboSAM eluted from the C8 column later than its unmodified counterpart (Figure S4A,B). In the urea-PAGE, 37Cy3-74Cy5-riboSAM migrated slower than the unmodified riboSAM (Figure S4C). Additionally, 37Cy3-74Cy5-riboSAM was visible under fluorescence excitation at both 550 nm and 650 nm during HPLC and PAGE (Figure S4B,C). The observed longer retention time in the C8 column, slower migration in urea-PAGE, and detectable fluorescence were attributed to the hydrophobic, bulky, and fluorescent properties of Cy3 and Cy5, confirming the successful incorporation of Cy3 and Cy5 into riboSAM. Moreover, the presence of a single peak in HPLC and a single band in urea-PAGE demonstrated the high purity of the 37Cy3-74Cy5-riboSAM sample. Hybridization of the 3′ end of 37Cy3-74Cy5-riboSAM with a 12-nt biotin-modified DNA tether enabled the immobilization of the biotin-DNA/37Cy3-74Cy5-riboSAM complex onto a polyethylene glycol (PEG)-passivated and streptavidin-coated quartz slide for smFRET measurements (Figure 1B,C).

### 3.2. smFRET Detection Reveals Three Distinct States of riboSAM in the Absence of Mg^2+^ and SAM

**Figure 2:**
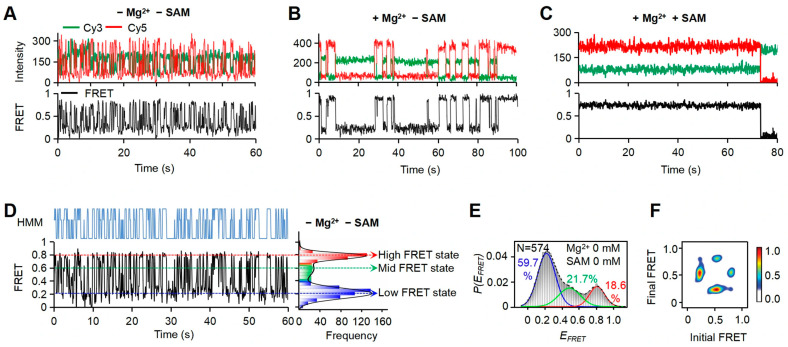
smFRET analysis of riboSAM under different conditions. (**A**–**C**) Representative single-molecule traces of 37Cy3-74Cy5-riboSAM in the absence of Mg^2+^ and SAM (**A**), in the presence of only Mg^2+^ (**B**), and in the presence of both Mg^2+^ and SAM (**C**). (**D**) Example smFRET trajectory exhibiting dynamic transitions among three FRET states (observed in almost all of the recorded traces in the absence of Mg^2+^ and SAM). Time-dependent *E_FRET_* changes were displayed as the black trajectory. The HMM fit was shown as a blue line above the trajectory and the corresponding FRET distribution, fitted with three Gaussian peaks (black curve), was presented on the right. (**E**) The *E_FRET_* histogram was generated from the HMM traces of 574 individual molecules in the absence of Mg^2+^ and SAM. The histogram was well fitted with three Gaussian peaks, shown in blue (*E_FRET_* ~0.2), green (*E_FRET_* ~0.6), and red solid lines (*E_FRET_* ~0.8), respectively, with their cumulative distribution displayed as a black dashed line. The fractional population of all fitted peaks was reported in respective colors in each histogram panel, and the number of molecules that were analyzed is indicated by ‘N’. (**F**) TODP corresponding to the histogram in (**E**). TODP displays dynamic traces as ‘off-diagonal’ and static traces as ‘on-diagonal’ features, where the color scale quantifies the prevalence of each population.

To address the effects of both Mg^2+^ and SAM on the conformation switches of riboSAM, we used smFRET to monitor the structural dynamics of 37Cy3-74Cy5-riboSAM under different Mg^2+^ and SAM conditions. Based on statistical analysis of the time-dependent FRET efficiency (*E_FRET_*) trace for individual molecules and Hidden Markov Model (HMM) analysis, we identified three distinct FRET states for 37Cy3-74Cy5-riboSAM with low- (~0.2), mid- (~0.6), and high-*E_FRET_* (~0.8) in the absence of Mg^2+^ and SAM (Figure 2A,D). These states correspond to the previously observed *apo*, *transit-p*, and *holo-p* conformations, respectively [20]. To quantify the relative populations of these three conformations, we generated an *E_FRET_* histogram from 574 individual 37Cy3-74Cy5-riboSAM molecules and analyzed it by fitting with a sum of multiple Gaussian peaks. Consistent with the HMM trace of individual molecules, the *E_FRET_* histogram was best fitted with three peaks centered at *E_FRET_* of ~0.2, ~0.6, and ~0.8, respectively (Figure 2E). The proportions of these three states were ~59.7% (~0.2), ~21.7% (~0.6), and ~18.6% (~0.8), indicating that the majority of molecules are in the *apo* state in the absence of Mg^2+^ and SAM.

To visualize the dynamic behavior of 37Cy3-74Cy5-riboSAM, we generated a transition occupancy density plot (TODP) from HMM traces of the 574 individual molecules. The TODP displays the fraction of single-molecule traces that exhibit at least once for any given initial to final FRET transition as a heat map at off-diagonal, while simultaneously displaying static trajectories along the diagonal [38]. Both the single-molecule traces and TODP indicated that 37Cy3-74Cy5-riboSAM dynamically interconverts among FRET states in the absence of Mg^2+^ and SAM, as indicated by the off-diagonal contours in the TODP (Figure 2F). Notably, the most frequent switches occurred between the *E_FRET_* ~0.2 and ~0.6 states, followed by transitions between the *E_FRET_* ~0.8 and ~0.6 states. Direct transitions between the *E_FRET_* ~0.2 and ~0.8 states were relatively rare. These observations further support the idea that the *transit-p* conformation serves as a crucial intermediate state bridging the *apo* and *holo-p* conformations, and riboSAM undergoes a sequential folding pathway comprising an initial transition from *apo* to the *transit-p* intermediate, followed by subsequent folding into the *holo-p* conformation. Upon the addition of Mg^2+^ and SAM, we observed a marked reduction in riboSAM’s dynamics, with the majority of molecules folding into the stable *holo-p* state (Figure 2B,C).

### 3.3. Increasing Mg^2+^ Induces the Folding of riboSAM into Two Distinct holo-p States, First Dynamic, Then Static

**Figure 3:**
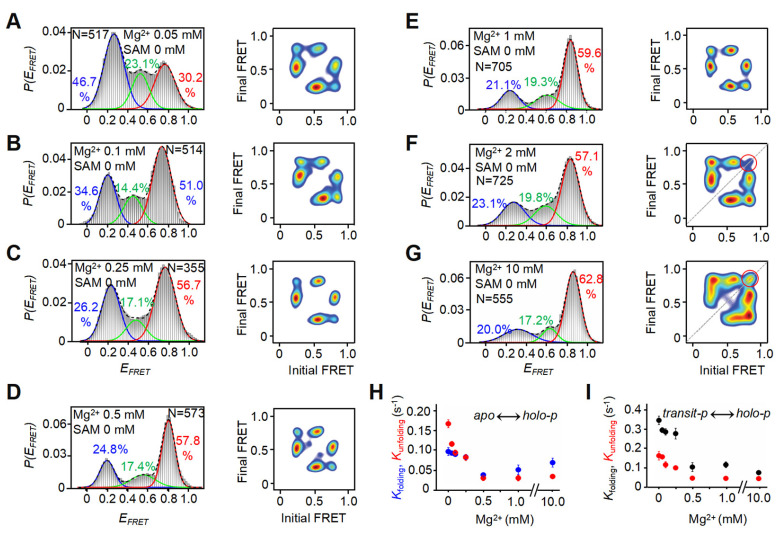
Effects of Mg^2+^ on riboSAM dynamic transitions. (**A**–**G**) *E_FRET_* histograms (left) and corresponding TODPs (right) of 37Cy3-74Cy5-riboSAM in the presence of 0.05 mM (**A**), 0.1 mM (**B**), 0.25 mM (**C**), 0.5 mM (**D**), 1 mM (**E**), 2 mM (**F**), and 10 mM Mg^2+^ (**G**), respectively. The red circles highlighted in the TODPs (F and G) mark the stable population of *holo-p* state maintaining an *E_FRET_* of 0.8. (**H**,**I**) Transition kinetics of *apo* to *holo-p* (**H**) and *transit-p* to *holo-p* (**I**) as a function of Mg^2+^ concentration. Mean ± SD values of triplicate experiments are shown.

To further elucidate the role of Mg^2+^ in riboSAM folding dynamics, we characterized the FRET signatures of 37Cy3-74Cy5-riboSAM across a range of Mg^2+^ concentrations. By statistically analyzing 300–800 single-molecule trajectories, we generated *E_FRET_* histograms and performed multi-Gaussian peak fitting. Similar to the FRET states observed in the absence of Mg^2+^ and SAM, we found *E_FRET_* values of ~0.2, 0.6, and 0.8 in the presence of 0.05–10 mM Mg^2+^. However, as the Mg^2+^ increased, the low-*E_FRET_ apo* conformation gradually decreased, from ~59.7% in the absence of Mg^2+^ to ~34.6% at 0.1 mM Mg^2+^ and eventually saturating at ~21.1% at 1 mM Mg^2+^ (Figure 2E and Figure 3A–G). In contrast, the high-*E_FRET_ holo-p* conformation increased from ~18.6% in the absence of Mg^2+^ to ~51.0% at 0.1 mM Mg^2+^ and eventually reaching an equilibrium of ~59.6% at 1 mM Mg^2+^ (Figure 2E and Figure 3A–G). These results demonstrate that Mg^2+^ ions induce riboSAM to fold into the *holo-p* state. This Mg^2+^-dependent shift in conformational distribution followed a Hill equation fit, yielding a dissociation constant *K_d_* ~0.06 mM for Mg^2+^ binding to riboSAM (Figure S5). Given that the Mg^2+^ concentration in bacteria ranges from 0.5 to 4 mM [22,23,27], this finding suggests that physiological level of Mg^2+^ promote the folding of riboSAM into the *holo-p* state.

**Figure 4:**
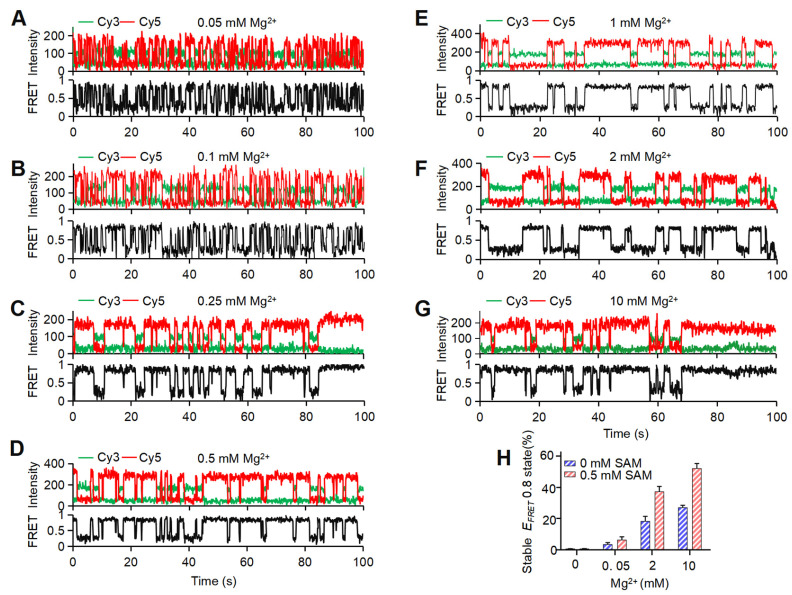
Gradually increasing Mg^2+^ concentrations reduce the dynamics of riboSAM. (**A**–**G**) Representative single-molecule traces for 37Cy3-74Cy5-riboSAM in the presence of 0.05 mM (**A**), 0.1 mM (**B**), 0.25 mM (**C**), 0.5 mM (**D**), 1 mM (**E**), 2 mM (**F**), and 10 mM Mg^2+^ (**G**), respectively. Time-dependent *E_FRET_* changes were displayed as the black trajectories. (**H**) Percentage of all traces that remain static in the high-*E_FRET_* state as a function of Mg^2+^ concentration in the absence (blue, *holo-p* state) and presence (red, *holo* state) of 0.5 mM SAM. Mean ± SD values of triplicate experiments are shown.

Single-molecule trajectories and TODPs further revealed that riboSAM still dynamically transitions among different FRET states in the presence of Mg^2+^. However, as the Mg^2+^ concentration increases, the molecules gradually stabilize, exhibiting significantly reduced transition frequencies between the three states (Figure 2A and Figure 4A–G). Particularly, in the presence of 2 mM and 10 mM Mg^2+^, a sub-population of static traces in the high-*E_FRET_* (*holo-p*) emerges, as evidenced by the *E_FRET_* ~0.8 peak on the diagonal in the TODP (Figure 3F,G, red circle). Statistical analysis showed that, at 0.05 mM Mg^2+^, only ~3.6% of the traces adopt this stable *holo-p* state, rising to ~18.5% and 27.4% at 2 mM and 10 mM Mg^2+^, respectively (Figure 4H and Table S3). Taken together, these data demonstrate that, in the absence of Mg^2+^, riboSAM primarily adopts the *apo* conformation while exhibiting pronounced structural dynamics characterized by transient shifts to the *transit-p* and *holo-p* states. Increasing the Mg^2+^ concentration induces folding of riboSAM, initially into the dynamic *holo-p* state, with a sub-population of molecules progressively stabilizing into the stable *holo-p* conformation at elevated Mg^2+^ concentrations (2 mM and 10 mM).

Except for a minimal population in the stable *holo-p* state, a significant fraction of molecules maintain a dynamic transition between different FRET states, as demonstrated by the off-diagonal contours in the TODPs (Figure 3F,G). Based on HMM analysis, we calculated the transition rates between the three distinct conformations. For *apo* to *holo-p*, the approximate rate constants for folding (*k*_foldin_*_g_*) and unfolding (*k*_unfolding_) are 0.097 S^−1^ and 0.167 S^−1^ in the absence of Mg^2+^, slowing to 0.051 S^−1^ and 0.030 S^−1^, respectively, in the presence of 1 mM Mg^2+^ (Figure 3H, and Table S4). The pronounced decrease in the *k*_unfolding_ value demonstrated that Mg^2+^ stabilizes riboSAM, promoting formation of the *holo-p* conformation. Above 1 mM Mg^2+^, the transition rates remain constant, indicating that physiological Mg^2+^ concentrations effectively induce riboSAM to fold into the dynamic *holo-p* state, while higher Mg^2+^ concentrations (2 mM and 10 mM) are necessary to drive the formation of the stable *holo-p* conformation. A similar trend was observed for the rates of folding and unfolding between *transit-p* and *holo-p* (Figure 3I, and Table S4). Notably, when the Mg^2+^ concentration was low, transition from *transit-p* to *holo-p* (*k*_foldin_*_g_*) occurred at a significantly higher rate than other transition processes, further supporting the role of the *transit-p* as a crucial intermediate that preferentially converts to *apo* and *holo-p* states.

### 3.4. SAM Locks the Mg^2+^-Induced Dynamic holo-p to Stable holo State by Conformational Selection

**Figure 5:**
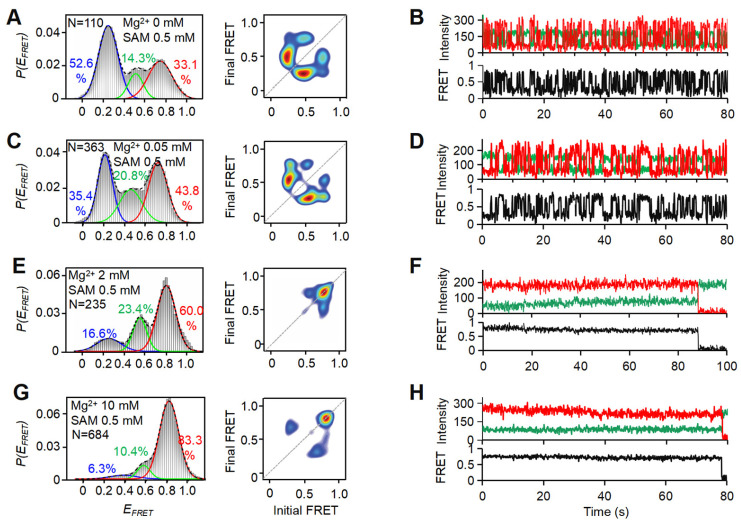
Effects of SAM on the folding of riboSAM. (**A**,**C**,**E**,**G**) *E_FRET_* histograms (left) and corresponding TODPs (right) of 37Cy3-74Cy5-riboSAM in the presence of 0.5 mM SAM and 0 mM (**A**), 0.05 mM (**C**), 2 mM (**E**), and 10 mM Mg^2+^ (**G**), respectively. (**B**,**D**,**F**,**H**) Representative single-molecule traces for 37Cy3-74Cy5-riboSAM in the presence of 0.5 mM SAM and 0 mM (**B**), 0.05 mM (**D**), 2 mM (**F**), and 10 mM Mg^2+^ (**H**), respectively.

Next, we sought to examine how riboSAM responds to the binding of its ligand, SAM. Previous studies revealed that 0.1–0.5 mM SAM are sufficient to trigger ligand-dependent conformational switches of riboSAM [20]. Based on this observation, we introduced 0.5 mM SAM to examine the ligand-induced dynamic conformational transitions of 37Cy3-74Cy5-riboSAM. In the absence of Mg^2+^ or with only 0.05 mM Mg^2+^ present, the addition of 0.5 mM SAM did not induce significant structural transitions in riboSAM (Figure 5A). Apart from a slight increase (~15%) in the population of the *E_FRET_* ~0.8 state (*holo*) in the FRET distribution, there were no changes in the TODPs or single-molecule trajectories (Figure 5A–D). These findings suggested that SAM binding to the RNA is remarkably weak under Mg^2+^-free or extremely low Mg^2+^ concentration conditions, failing to induce substantial conformational changes. However, in the presence of 2 mM Mg^2+^, the addition of 0.5 mM SAM promoted riboSAM folding into the stable *transit* and *holo* conformations, as evidenced by *E_FRET_* ~0.6 and ~0.8 peaks along the diagonal in the TODP, as well as stable *E_FRET_* ~0.6 and 0.8 states in single-molecule trajectories (Figure 5E,F and Figure S6). This indicated that SAM locks the Mg^2+^-induced dynamic *transit-p* and *holo-p* into stable *transit* and *holo* states. Increasing Mg^2+^ to 10 mM shifted the majority of molecules into the stable *holo* conformation (Figure 5G,H). Statistical analysis of the HMM data further revealed that the stable *E_FRET_* ~0.8 population increased from ~18.5% at 2 mM Mg^2+^ alone to ~37.6% with 2 mM Mg^2+^ and 0.5 mM SAM, and from ~27.4% at 10 mM Mg^2+^ alone to ~52.6% with 10 mM Mg^2+^ and 0.5 mM SAM (Figure 4H and Table S3). These findings confirmed that SAM stabilizes the Mg^2+^-induced dynamic *holo-p* conformation.

## 4. Discussion

**Figure 6:**
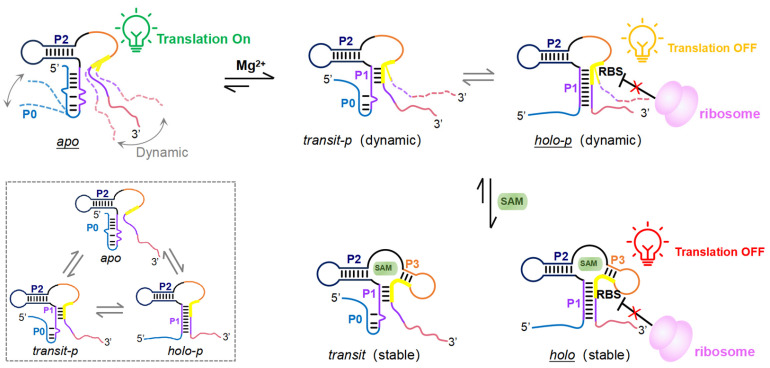
Model for Mg^2+^- and SAM-dependent translation regulation of riboSAM. The predominant structures under different Mg^2+^ and SAM conditions are underlined. The RBS is shown as a bold yellow line. In the absence of both Mg^2+^ and SAM, the *apo* state predominates, while highly dynamic transitions among the *apo*, *transit-p*, and *holo-p* states are shown in the dotted box. Increasing Mg^2+^ induced riboSAM to fold into dynamic *transit-p* and *holo-p* states, particularly the *holo-p* state. SAM binds and stabilizes the dynamic *transit-p* and *holo-p* states, locking them into the stable *transit* and *holo* states to turn off downstream gene expression strictly.

Divalent cations play a pivotal role in the formation and stabilization of the 3D structure of RNA molecules. The riboswitch function depends on Mg^2+^-mediated folding into precise 3D conformations that are competent for ligand binding. Crystallographic studies of numerous riboswitches have revealed many of them adopt highly organized globular architectures with Mg^2+^-stabilized structural cores. These metal ion cores, analogous to hydrophobic cores in proteins, facilitate proper RNA tertiary folding, as exemplified by the aptamer domains of purine riboswitches, which recognize adenine or guanine [30]. Similarly, high-resolution X-ray crystallographic analysis of the SAM-VI riboswitch in its ligand-bound state showed the presence of multiple well-defined Mg^2+^ coordination sites [19]. Our previous riboSAM co-transcriptional folding studies, conducted in Mg^2+^-dependent systems required for RNA polymerase activity, failed to characterize its Mg^2+^-free structural properties or elucidate Mg^2+^’s role in tertiary structure formation and ligand binding [20]. In this study, we used site-specific fluorescence donor-acceptor labeling coupled with smFRET to elucidate the role of Mg^2+^ in the tertiary folding, ligand recognition, and dynamic structural transitions of the full-length riboSAM. This approach allowed us to precisely characterize the Mg^2+^- and SAM-responsive structural transitions at single-molecule resolution. Our findings demonstrated that, similar to the structures formed during transcription [20], the post-transcriptional riboSAM also adopts five distinct conformations—*apo*, *transit*/*transit-p*, and *holo*/*holo-p*—whose conformational transitions and dynamics are regulated by Mg^2+^ and SAM concentrations (Figure 6).

In the absence of Mg^2+^ and SAM, riboSAM predominantly adopted the *apo* conformation, exhibiting pronounced structural dynamics while transiently sampling folding intermediates that resemble the *transit-p* and *holo-p* states (Figure 6). Mg^2+^ was found to induce the folding of riboSAM into two distinct *holo-p* states: the dominant dynamic *holo-p* state and the minor stabilized folded *holo-p* state, which emerged at elevated Mg^2+^ concentrations (2 and 10 mM). This Mg^2+^ ions-mediated folding appears to generate a transient, structurally pliable binding pocket that serves as a conformational prerequisite for subsequent SAM recognition and binding. SAM then binds to this pocket, locking the dynamic *holo-p* into the final stable *holo* conformation (Figure 6). Within the framework of the two established molecular recognition paradigms—conformational selection (CS, “folding first”) and induced-fit (IF, “binding first”) [28,39,40]—our structure data demonstrated that SAM exclusively binds to the Mg^2+^-induced folded conformation of riboSAM, thus following the CS mechanism. This leads to the formation of a thermodynamically stable three-way junction structure that ultimately allosterically represses the translation of the downstream SAM synthetase gene. This riboSAM-mediated negative feedback circuit enables precise homeostatic control of cellular SAM concentration. The single-molecule dynamic structural measurements in this study provide critical insights into how Mg^2+^ and SAM drive riboSAM conformational transitions. These findings significantly advance our mechanistic understanding of how riboswitches regulate gene expression by responding to environmental signals through dynamic structural switches.

## 5. Conclusions

Mg^2+^ plays a pivotal role in RNA folding and the formation of intricate three-dimensional structures. Extensive studies have established that Mg^2+^ promotes numerous riboswitches to fold into compact and docking-competent conformations, which serve as essential prerequisites for subsequent ligand recognition through the CS mechanism. As a novel class within the SAM-responsive riboswitch family, the dynamic structural mechanisms governing gene regulation by riboSAM warrant systematic investigation. In our work, the complex conformational transitions of riboSAM in response to Mg^2+^ and the ligand SAM were precisely captured at the single-molecule level. We demonstrated that Mg^2+^ ions induce riboSAM to fold into specific three-dimensional structures, creating a favorable pocket for ligand binding. SAM then binds to this pocket through a CS mechanism and locks riboSAM into its final stable conformation. This synergistic regulation by Mg^2+^ and SAM in promoting riboSAM folding and dynamic structural transitions, which remained undiscovered in previous co-transcriptional detections, provides a new perspective on the mechanism by which riboSAM regulates gene expression in response to ligand. And these findings provide new insights into the gene regulatory mechanism of riboswitches, establishing a paradigm for elucidating dynamic conformational switches within broader riboswitch families. Moreover, the clarification of the conformational switching pattern of riboSAM will provide a structural basis for the design of antibacterial drugs and SAM-biosensors targeting this riboswitch, as well as for the development of riboSAM as a synthetic biology element [41,42,43,44,45].

## Data Availability

The data presented in this study are available in insert article or Supplementary Materials here.

## Supplementary Materials

The following supporting information can be downloaded at: https://www.mdpi.com/article/10.3390/biom15060841/s1, Figure S1: The secondary structures of *apo*, *transit-p*, *transit* (*transit-p* binds with SAM), *holo-p*, and *holo* (*holo-p* binds with SAM) states of riboSAM; Figure S2: The chemical structures of Cy3-UTP (A) and Cy5-UTP (B); Figure S3: The schematic procedure of 12-step PLOR for 37Cy3-74Cy5-riboSAM synthesis; Figure S4: Characterization of 37Cy3-74Cy5-riboSAM via RP-HPLC and urea-PAGE; Figure S5: The proportions of the *E_FRET_* ~0.2 (A) and *E_FRET_* ~0.8 states (B) are plotted as a function of Mg^2+^ concentration, yielding the dissociation constant *K_d_* value; Figure S6: The representative single-molecule traces (*E_FRET_* ~0.6) for 37Cy3-74Cy5-riboSAM in the presence of both 2 mM Mg^2+^ and 0.5 mM SAM; Table S1: The DNA/RNA sequences used for riboSAM synthesis; Table S2: Reagents usage in 12 step-PLOR to generate 37Cy3-74Cy5-riboSAM; Table S3: Percentage of the stable high-FRET state (*E_FRET_* ~0.8) under different Mg^2+^ and SAM concentrations; Table S4: Observed transition rates.

## Abbreviations

The following abbreviations are used in this manuscript:

PLOR	position-selective labeling of RNA
smFRET	single-molecule Förster resonance energy transfer
RP-HPLC	reverse-phase high-performance liquid chromatography
HMM	hidden Markov modeling
TODP	transition occupancy density plot
