# Supplementary material for: Observation of SAM-VI Riboswitch Dynamics Using Single-Molecule FRET
Source: Biomolecules. 2025 Jun 9;15(6):841. doi: 10.3390/biom15060841 (PMC12190640; doi:10.3390/biom15060841)
Supplement: Supplementary file 1 [file biomolecules-15-00841-s001.zip › biomolecules-3660045-supplementary.pdf]

Supplementary Information for

Observation of SAM-VI riboswitch dynamics using single-molecule FRET

Yanyan Xue [1, 2, 3]*, Yi Sun [1], Yichun Xia [1], Xiuming Liu[1] and Hua Dai [1, 2]*

[1] Institute of Translational Medicine, School of Medicine, Yangzhou University, Yangzhou, 225001, China; 17315355815@163.com (Y.S.); 18762990859@163.com (Y.C.X.); 13851110481@163.com (X.L.)

[2] The Key Laboratory of the Jiangsu Higher Education Institutions for Nucleic Acid & Cell Fate Regulation (Yangzhou University), Yangzhou, 225001, China;

[3] State Key Laboratory of Microbial Metabolism, School of Life Science and Biotechnology, Shanghai Jiao Tong University, Shanghai, 200240, China;

* Correspondence: yanyanxue@yzu.edu.cn; daihua@yzu.edu.cn;

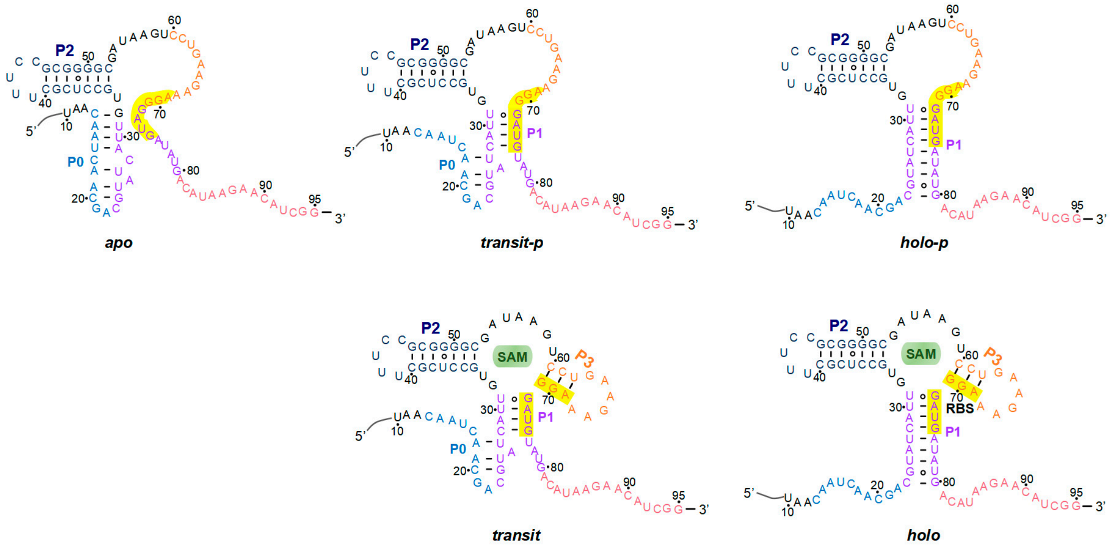

Figure S1. The secondary structures of *apo*, *transit-p*, *transit* (*transit-p* binds with SAM), *holo-p*, and *holo* (*holo-p* binds with SAM) states of riboSAM**.**

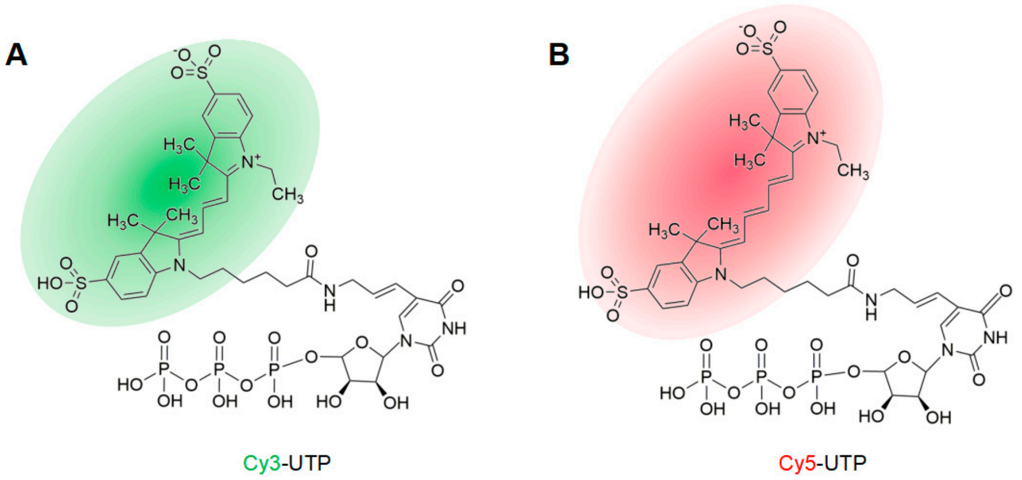

Figure S2. The chemical structures of Cy3-UTP (**A**) and Cy5-UTP (**B**).

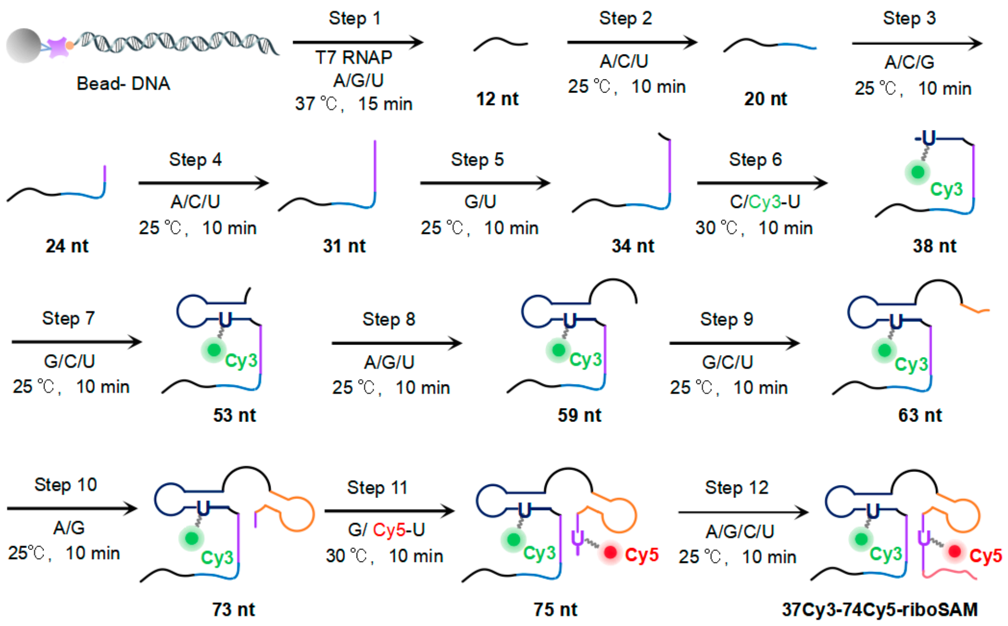

Figure S3. The schematic procedure of 12-step PLOR for 37Cy3-74Cy5-riboSAM synthesis.

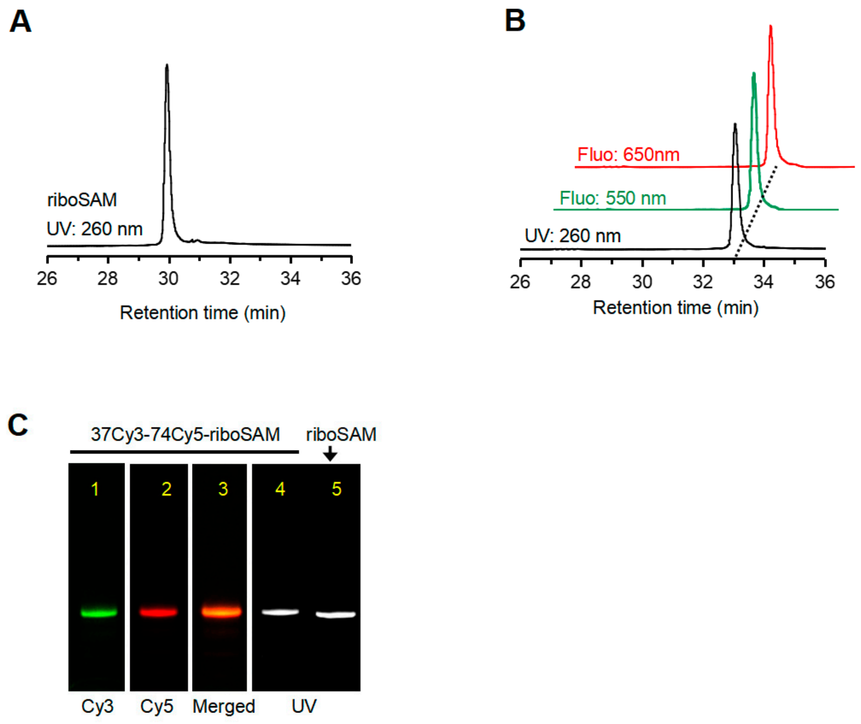

Figure S4. Characterization of 37Cy3-74Cy5-riboSAM via RP-HPLC and urea-PAGE. **(A)** HPLC spectrum of unlabeled riboSAM at UV (260 nm) irridiation. **(B)** HPLC spectra of 37Cy3-74Cy5-riboSAM with 260 nm UV irradiation (in black), 550 nm fluorescence excitation (in green) and 650 nm fluorescence excitation (in red). **(C)** PAGE images of 37Cy3-74Cy5-riboSAM under fluorescent and UV irradiation. The lanes 1 and 2 were irradiated under 530 nm and 620 nm fluorescence, respectively. The lane 3 is the merged image of the lanes 1 and 2. Unlabeled riboSAM was loaded at lane 5 as a control.

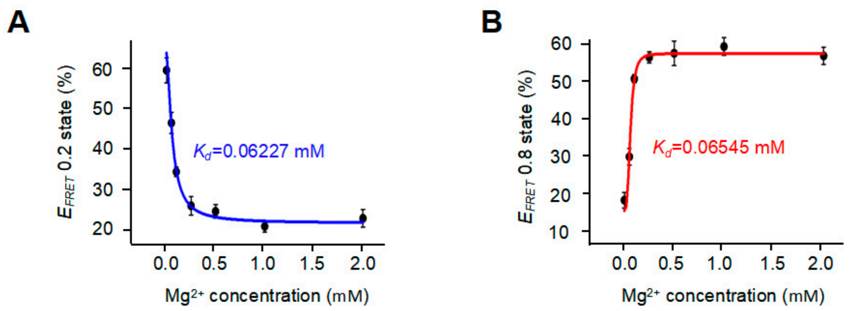

Figure S5. The proportions of the E_{FRET} ~ 0.2 (**A**) and E_{FRET} ~ 0.8 states (**B**) are plotted as a function of Mg^{2+} concentration, yielding the dissociation constant K_d value. Mean ± SD values of triplicate experiments are shown.

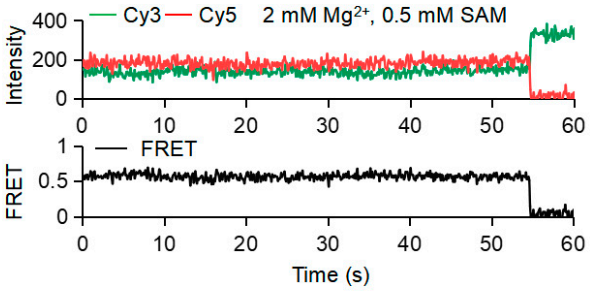

Figure S6. The representative single-molecule traces (E_{FRET} ~ 0.6) for 37Cy3-74Cy5-riboSAM in the presence of both 2 mM Mg^{2+} and 0.5 mM SAM.

Table S1. The DNA/RNA sequences used for riboSAM synthesis

DNA/RNA	Sequence
riboSAM	5′-GGGAAGAAAUAACAAUCAACGACGUAUCAUU<br>GUGCCUCGCUUUCCGCGGGGCGAUAAGUCCUGA<br>AGAAAGGGAUGAUAUGACAUAAGAACAUCGG
DNA template strand for riboSAM	5′-mCmCGATGTTCTTATGTCATATCATCCCTTTCT<br>TCAGGACTTATCGCCCCGCGGAAAGCGAGGCAC<br>AATGATACGTCGTTGATTGTTATTTCTTCCC<u>TATA</u><br><u>GTGAGTCGTATTA</u>*TGGACTAGCTGAATCAGA*
DNA coding strand for riboSAM	5′-biotin-*TCTGATTCAGCTAGTCCA*<u>TAATACGACTCA</u><br><u>CTATA</u>GGGAAGAAATAACAATCAACGACGTATC<br>ATTGTGCCTCGCTTTCCGCGGGGCGATAAGTCCT<br>GAAGAAAGGGATGATATGACATAAGAACATCGG
Forward primer for riboSAM-DNA PCR	5’-biotin-TCTGATTCAGCTAGTCCATAATACGACT
Reverse primer for riboSAM-DNA PCR	5′-mCmCGATGTTCTTATGTCATATCATCCCTT
12 nt DNA for hybridization with riboSAM in smFRET	5′-biotin-CCGATGTTCTTA

The T7 promoter sequences in DNAs are underlined, and the linker sequences (italic) are inserted in DNA to alleviate potential steric hindrance between beads and T7 RNAP during transcription. mC represents 2′-O-methyl-dC.

Table S2. Reagents usage in 12 step-PLOR to generate 37Cy3-74Cy5-riboSAM

Reagent usage (10 μM, 1 mL)
**Step 1** in the buffer (6 mM $MgSO_4$, 40 mM Tris-HCl, 100 mM K_2SO_4, 10 mM DTT, pH 8.0) at 37 ºC for 15 min:<br>10 μM DNA-beads, 10 μM T7 RNAP, 1.12 mM ATP, 0.96 mM GTP and 32 μM UTP;<br>**Steps 2–12** in the buffer (6 mM MgSO4, 40 mM Tris-HCl, 10 mM DTT, pH 8.0) at 25 or 30 ºC for 10 min:<br>**Step 2:** 40 μM ATP, 30 μM:CTP, 10 μM UTP;<br>**Step 3:** 10 μM ATP, 10 μM CTP, 20 μM GTP;<br>**Step 4:** 20 μM ATP, 10 μM CTP, 40 μM UTP;<br>**Step 5:** 20 μM GTP, 10 μM UTP;<br>**Step 6:** 30 μM CTP, 10 μM Cy3-UTP (30 ºC, 10 min);<br>**Step 7:** 50 μM CTP, 70 μM GTP, 30 μM UTP;<br>**Step 8:** 30 μM ATP, 10 μM GTP, 20 μM UTP;<br>**Step 9:** 10 μM GTP, 20 μM CTP, 10 μM UTP;<br>**Step 10:** 60 μM ATP, 40 μM GTP;<br>**Step 11:** 10 μM GTP, 10 μM Cy5-UTP (30 ºC, 10 min);<br>**Step 12:** 90 μM ATP, 30 μM CTP, 40 μM GTP, 40 μM UTP.

Table S3. Percentage of the stable high-FRET state (E_{FRET} ~ 0.8) under different Mg^{2+} and SAM concentrations

Mg^{2+} (mM)	SAM (mM)	% E_{FRET} ~ 0.8 state
0	0	0.845
	0.5	0.862
0.05	0	3.630
	0.5	6.585
2	0	18.525
	0.5	37.600
10	0	27.390
	0.5	52.550

Table S4. Observed transition rates

	apo to *holo-p*		*transit-p* to *holo-p*	
Mg^{2+} (mM)	k_{folding}	$K_{un\text{folding}}$	k_{folding}	$K_{un\text{folding}}$
0	0.097	0.167	0.341	0.157
0.05	0.093	0.116	0.290	0.151
0.1	0.090	0.094	0.281	0.110
0.25	0.083	0.082	0.272	0.094
0.5	0.038	0.030	0.098	0.040
1	0.051	0.030	0.110	0.039
10	0.069	0.034	0.069	0.038
